# Interferon-γ-response to mycobacterial glycopeptidolipids in patients with pulmonary non-tuberculous mycobacterial infection

**DOI:** 10.1007/s00430-026-00892-0

**Published:** 2026-09-23

**Authors:** Mathis Steindor, Sophia Dachmann, Florian Stehling, Margarete Olivier, Jan Kehrmann, Peter A Horn, Raquel Villar-Hernández, Svenja Straßburg, Matthias Welsner, Sivagurunathan Sutharsan, Monika Lindemann

**Affiliations:** 1 Pediatric Pulmonology and Sleep Medicine, Children’s University Hospital Essen, University of Duisburg-Essen, Essen, Germany; 2 Department of General Pediatrics, Neonatology and Pediatric Cardiology, Medical Faculty, University Hospital Düsseldorf, Heinrich Heine University, Düsseldorf, Germany; 3 Institute of Medical Microbiology, University Hospital Essen, University of Duisburg-Essen, Essen, Germany; 4 Institute for Transfusion Medicine, University Hospital Essen, University of Duisburg-Essen, Essen, Germany; 5 Genome Identification Diagnostics (GenID) GmbH, Straßberg, Germany; 6 Department of Pulmonary Medicine, Adult Cystic Fibrosis Center, University Hospital Essen- Ruhrlandklinik, University of Duisburg-Essen, Essen, Germany

**Keywords:** Non-tuberculous mycobacteria, Cystic fibrosis, Diagnostic tool, Pulmonary disease, Immune response

## Abstract

The understanding of host responses to pulmonary infection with non-tuberculous mycobacteria (NTM) is incomplete and immunodiagnostic tools are not established. Unlike mycobacteria of the *Mycobacterium tuberculosis* complex, several NTM express glycopeptidolipids (GPLs) in their cell wall. Here, we investigate the interferon-γ response to mycobacterial GPLs and its diagnostic use in patients with pulmonary NTM infection. *M. avium*-derived GPLs were used for in vitro stimulation of peripheral blood mononuclear cells in an ELISpot assay of patients with pulmonary NTM infection and 3 control groups, (1) patients with tuberculosis (TB), (2) patients with cystic fibrosis without evidence of NTM infection (CF) and (3) healthy controls (HC). Immune responses were compared between the groups and for the subgroup of NTM patients with either *M. avium* complex (MAC) or *M. abscessus* complex (MABC). Interferon-γ responses of 31 patients with NTM (16 MABC, 11 MAC, 1 MABC & MAC, and 3 other NTM) were compared to 43 controls (15 TB, 15 CF, 13 HC). Responses to GPLs were significantly stronger in the NTM group compared to controls (13.5 vs. 1.5 spots per 300,000 PBMC, *p* < 0.0001, data represent median values). MAC patients had an overall stronger reaction than MABC patients. Assay sensitivity and specificity were 64 and 98% for MAC, 19 and 95% for MABC, and 32 and 98% for all NTM, respectively. Mycobacterial GPLs are potent drivers of interferon-γ-responses. Patients with pulmonary NTM infection show stronger immune responses to GPLs compared to uninfected participants, making GPLs a promising target for the development of NTM immune diagnostic tools.

## Introduction

Pulmonary infections caused by non-tuberculous mycobacteria (NTM) - including *Mycobacterium avium* complex (MAC) and *Mycobacterium abscessus* complex (MABC) - pose a serious clinical challenge, especially in individuals with chronic lung diseases such as cystic fibrosis (CF) [1, 2]. Diagnosing NTM pulmonary disease (NTM-PD) remains difficult, and although interferon-γ release assays (IGRAs) have revolutionized tuberculosis diagnostics, to this date, they do not play a significant role in NTM disease [3].

In contrast to *Mycobacterium tuberculosis*, many NTM species express glycopeptidolipids (GPLs) - surface-exposed glycolipids believed to modulate host-pathogen interactions and immune responses [4, 5]. Measuring anti-GPL-core IgA levels has shown moderate utility as a serologic marker for NTM pulmonary disease [6]. GPL-induced interferon-γ (IFN-γ) release has yielded promising results in distinguishing NTM-lymphadenitis from lymphadenitis of other causes, tuberculosis, and healthy controls in paediatric populations [7]. However, the GPL-driven IFN-γ response in pulmonary NTM infections has not yet been investigated.

In this study, we aimed to assess the diagnostic potential of a GPL-based IGRA for distinguishing individuals with pulmonary NTM infection from uninfected controls. We hypothesized that GPL-specific T-cell responses can discriminate patients with pulmonary NTM infection from individuals without NTM infection and therefore may provide the basis for a novel immunodiagnostic assay.

## Methods

### Study participants and design

Study participants were recruited at the pediatric and adult pulmonology departments of the University Hospital Essen (Department of Pediatric Pulmonology and Sleep Medicine and Department of Pulmonary Medicine, Ruhrlandklinik, Essen, Germany, respectively). Participants with at least one bronchoalveolar lavage sample positive for NTM or two separate sputum samples positive for the same NTM species prior to study enrollment were assigned to the NTM group, including CF and non-CF participants. Control groups were: (1) CF participants without microbiological evidence for NTM infection (no prior NTM infection with at least one negative sputum sample for NTM in the year prior to study enrolment). (2) Participants with *M. tuberculosis*-IGRA confirmed current or prior tuberculosis (TB). (3) Healthy participants.

### NTM GPLs

GPLs, previously purified from *M. avium* serovar 4 according to a published procedure [7], were provided by Autoimmun Diagnostika GmbH (Straßberg, Germany). The diagnostic use of these GPLs is patented under the patent reference EP3803400B1.

### ELISpot assays

Twenty milliliters of heparinized blood were collected from each study participant and peripheral blood mononuclear cells (PBMC) were separated by Ficoll gradient centrifugation. Numbers of PBMC were determined by an automated hematology analyzer (XP 300, Sysmex, Norderstedt, Germany). Cell cultures of 300,000 PBMC were incubated with the ready-to-use GPLs provided by AID Autoimmun Diagnostika at 37 °C in AIM V™ medium Gibco, Grand Island, NY, United States) in a final volume of 200 µl. PBMC stimulated with phytohemagglutinin (1 µg/mL) served as positive controls and PBMC without stimulation as negative controls. As a first step, the cells were preincubated for 48 h in U bottom plates (BD Falcon, Nijmegen, Netherlands). Thereafter, the cells were incubated for a further 20 h in precoated ELISpot plates, using a standardized system to detect IFN-γ production (T-Track^®^ ELISpot kit, Lophius Biosciences, Regensburg, Germany). Negative (unstimulated) controls were subtracted from those of the GPL-stimulated cultures and ELISpot results and given as spots increment.

In parallel, we tested for immune reaction against bacteria of the *M. tuberculosis complex* (*M. tuberculosis*, *M. africanum*,* M. bovis*), using a commercially available ELISpot (T-SPOT.*TB*, Oxford Immunotech, Abingdon, UK) also responding to human infections with *M. leprae* and several environmental mycobacteria (e.g., *M. kansasii*,* M. marinum* or *M. szulgai*) [3]. We exactly followed the manufacturer’s instructions. In brief, 250,000 PBMC were cultured for 20 h at 37 °C in 150 µL AIM V™ medium and were stimulated with *M. tuberculosis*-specific peptide pools [early secretory antigenic target (ESAT-6) and culture filtrate protein-10 (CFP-10)] or with phytohemagglutinin (positive control). In parallel, PBMC were cultured without stimulation (negative control). Reactions of ≥ 6 spots increment towards the *M. tuberculosis*-specific peptide pools were considered as positive.

### Statistical analysis

Assuming no normal distribution of data, we performed pairwise comparisons of cellular in vitro responses in several groups of study participants with and without previous NTM infection by Mann-Whitney *U* test. Sensitivity and specificity were determined by receiver operating characteristic (ROC) curve analysis. Statistical analysis was performed with GraphPad Prism version 8.4.2.679 (GraphPad Software, San Diego, CA, USA).

## Results

### Participant enrolment and characteristics

We enrolled 31 patients with pulmonary NTM infection (16 MABC, 11 MAC, 1 MABC & MAC, and 3 other NTM) for the *NTM* group, and 43 individuals in the control groups (15 *TB*, 15 *CF*, 13 *HC*), respectively. Participant characteristics are given in Table 1. One patient had concomitant infection with MABC and MAC, so he was included in the pooled analyses, but excluded from analyses of the subgroups with MABC and MAC infection, respectively. From 17 patients with MABC isolation, information on solid agar colony morphotypes of the MABC isolates were available for 12 MABC patients: Six had a smooth (GPL-rich) colony morphotype, one had a rough (GPL-low) colony morphotype, five patients harbored isolates of both colony morphotypes. The NTM species identified in the three participants of the *other NTM* group were *M. szulgai*, *M. gordonae* and *M. xenopi*. All NTM-positive participants fulfilled the microbiological criteria for NTM-PD as defined by the American Thoracic Society [2], while clinical (including radiological) criteria were not met in 16 of 31 participants as evaluated by the attending physicians. Of the 15 TB patients, all of whom with positive T-SPOT.*TB*, 12 had a current active tuberculosis (10 pulmonary, 1 central nervous system, 1 abdominal), one had prior pulmonary tuberculosis and two had latent tuberculosis.

**Table 1:** Patient characteristics

Group	*n*	Female sex, *n* (%)	Age, years (mean, range)	CF, *n* (%)	NTM-PD, *n* (%)
NTM	31	14 (47%)	36, 10–80	25 (83%)	15 (48%)
MAC	11	6 (55%)	46, 11–80	7 (64%)	5 (45%)
MABC	16	5 (31%)	27, 10–53	15 (94%)	8 (50%)
MABC + MAC	1	0	61	1 (100%)	1 (100%)
Other NTM	3	3 (100%)	43, 29–59	2 (66%)	1 (33%)
TB	15	4 (27%)	30, 7–65	0	0
CF	15	8 (53%)	36, 15–75	15 (100%)	0
HC	13	7 (54%)	28, 22–56	0	0
Total	74	33 (45%)	33, 7–80	40 (54%)	15 (21%)

*NTM* non-tuberculous mycobacteria, *TB* Tuberculosis, *CF* Cystic Fibrosis, *HC* Healthy Controls, *NTM-PD* NTM pulmonary disease

### Performance of NTM-specific IGRA for detection of NTM infection

**Fig. 1:**
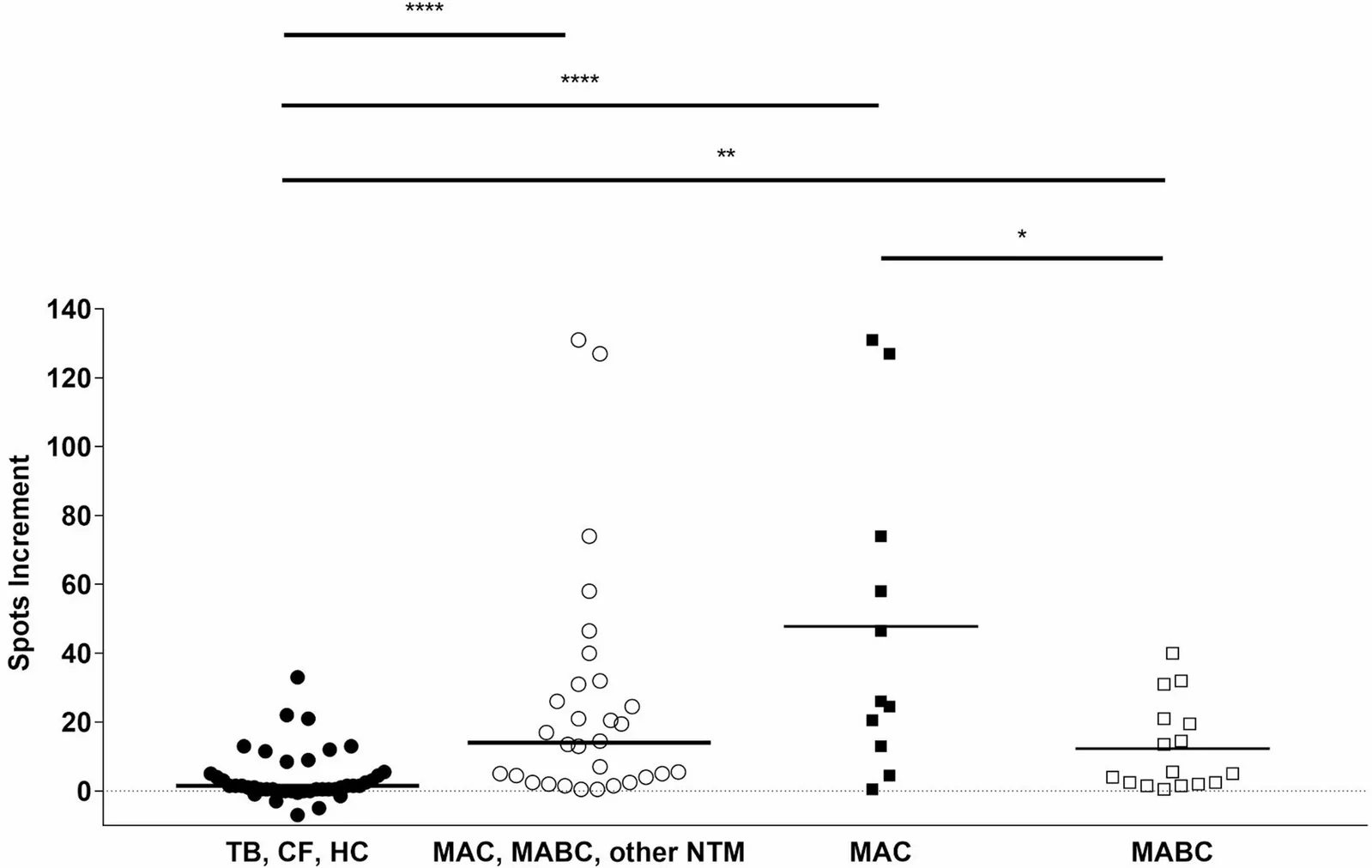
IFN-γ responses to glycopeptidolipids (GPLs) in pooled NTM patients (*n* = 31), the subgroup of MAC (*n* = 11) and MABC patients (*n* = 16), respectively, in comparison to pooled control groups (*n* = 43; TB, CF, HC) are shown as spots increment with median. Spots increment means GPL-stimulated cultures minus negative controls. *TB* Tuberculosis, *CF* Cystic Fibrosis, *HC* Healthy Controls, *MAC Mycobacterium avium* complex, *MABC Mycobacterium abscessus* complex, *NTM* non-tuberculous mycobacteria. * *p* < 0.05, **p < 0.01, ****p < 0.0001 (Mann-Whitney-U-Test)

**Fig. 2:**
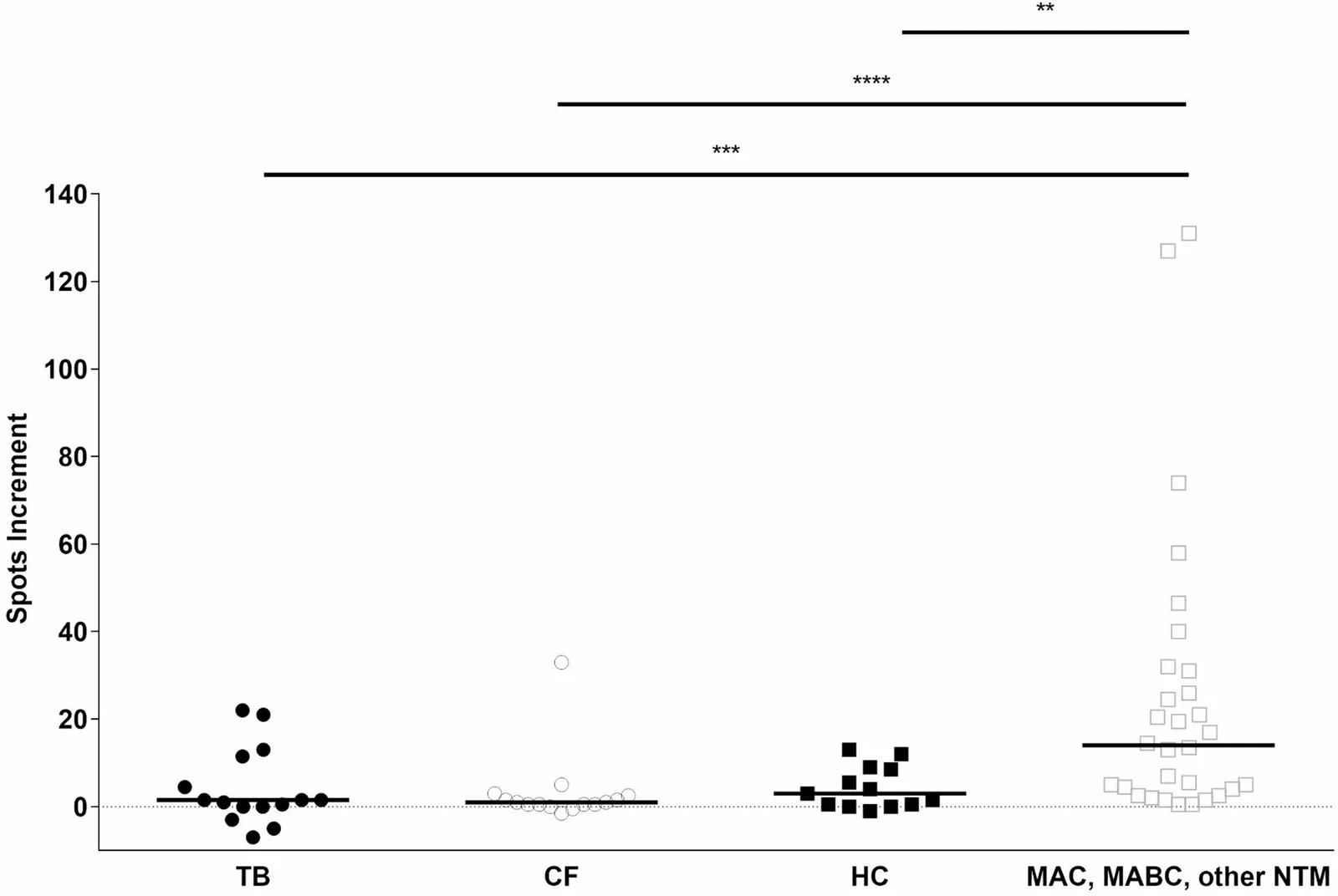
IFN-γ responses to glycopeptidolipids (GPLs) in all control groups (TB *n* = 15, CF *n* = 15, HC *n* = 13) in comparison to pooled NTM groups (*n* = 31) are shown as spots increment with median. Spots increment means GPL-stimulated cultures minus negative controls. *MAC Mycobacterium avium* complex, *MABC Mycobacterium abscessus* complex, *NTM* non-tuberculous mycobacteria, *TB* Tuberculosis, *CF* Cystic Fibrosis, *HC* Healthy Controls. **p < 0.01, ***p < 0.001, ****p < 0.0001 (Mann-Whitney-U-Test)

**Fig. 3:**
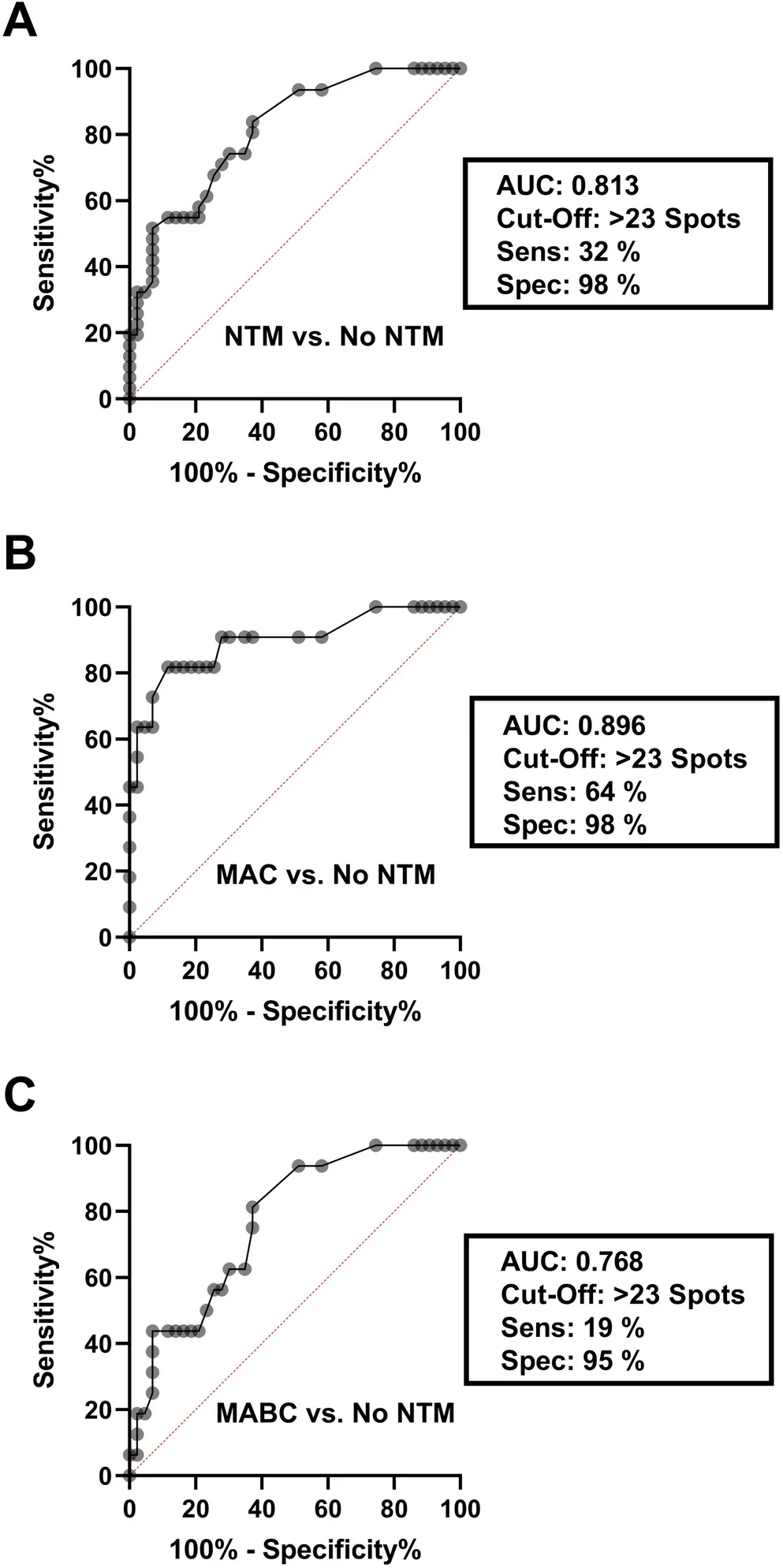
Results of receiver operating characteristic (ROC) curve analyses. It was analyzed whether ELISpot responses to the NTM GPLs were predictive of any NTM (panel A), MAC (panel B) or MABC (panel C) infection, respectively

**Fig. 4:**
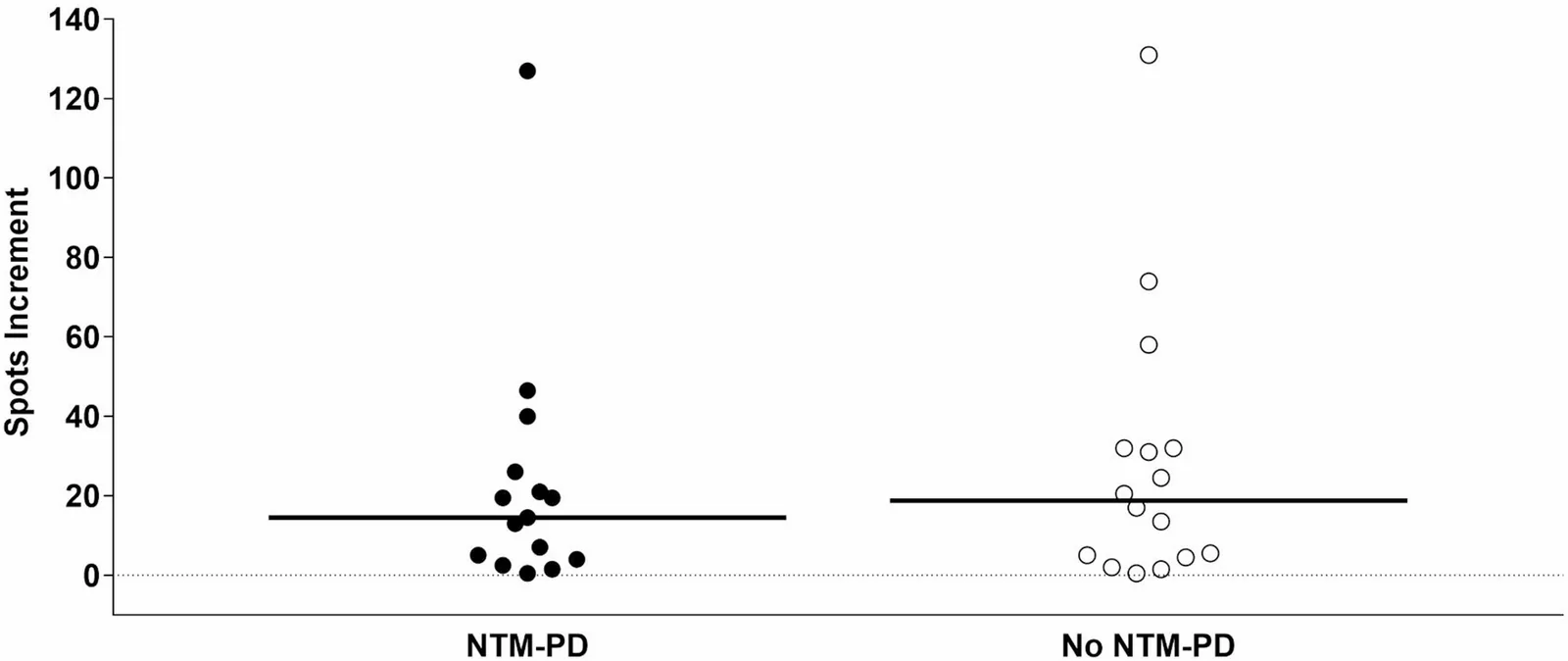
IFN-γ responses to glycopeptidolipids (GPLs), shown as spots increment with median, in patients with clinical NTM-PD (*n* = 15) as compared to patients with no clinical NTM-PD (*n* = 16). Spots increment means GPL-stimulated cultures minus negative controls. *NTM-PD* non-tuberculous mycobacterial pulmonary disease

A summary of the assay results according to NTM species and study group is provided in Table 2. Overall, cellular in vitro responses to GPLs were significantly stronger in the NTM group than in the pooled control groups (13.5 vs. 1.5 spots per 300,000 PBMC, *p* < 0.0001, data represent median values, Figs. 1, 2). Both the subgroups of MAC and MABC patients also had significantly stronger immune responses as compared to pooled controls (MAC: 26 vs. 1.5 spots, *p* < 0,0001, MABC 5.25 vs. 1.5 spots, *p* = 0.0012, Fig. 1). MAC patients had stronger immune responses than MABC patients (26 vs. 5.25 spots, *p* = 0.0205, Fig. 1). With an arbitrary cut-off for positive responses at > 23 spots increment, aiming at highest specificity while maintaining balance between sensitivity and specificity, the assay sensitivity and specificity were 64 and 98% for MAC, 19 and 95% for MABC, and 32 and 98% for all NTM respectively (Fig. 3). We did not find significant differences in cellular in vitro responses in patients labeled with clinical NTM-PD as compared to those with sole microbiological label of NTM-PD as determined by ATS criteria [2] (14.5 vs. 18.75 spots, *p* = 0.6328, Fig. 4. Additionally, pairwise comparisons revealed no statistically significant differences between cellular in vitro responses of MABC patients with mixed (*n* = 5, 13.5 spots) and rough (*n* = 1, 2.5 spots, *p* = 0.667), mixed and smooth (*n* = 6, 5 spots, *p* = 0.784), or rough and smooth (*p* = 0.801) colony morphotype isolates. Apart from the TB group, none of study participants had a positive T-SPOT.*TB* result (data not shown).

**Table 2:** Assay results according to NTM species and study group

Group / NTM species	*n*	Median spot increment	GPL-IGRA positive, *n* (%)	GPL-IGRA negative, *n* (%)
All NTM	31	13.5	10 (32%)	21 (68%)
MAC	11	26.0	7 (64%)	4 (36%)
MABC	16	5.25	3 (19%)	13 (81%)
MABC + MAC	1	1.5	0 (0%)	1 (100%)
Other NTM	3	7.0	0 (0%)	3 (100%)
└ *M. szulgai*	1	5.0	0 (0%)	1 (100%)
└ *M. gordonae*	1	17.0	0 (0%)	1 (100%)
└ *M. xenopi*	1	7.0	0 (0%)	1 (100%)
**All controls**	**43**	**1.5**	**1 (2%)**	**42 (98%)**
TB	15	1.5	0 (0%)	15 (100%)
CF	15	1.0	1 (7%)	14 (93%)
HC	13	3.0	0 (0%)	13 (100%)

Assay results were classified as positive using a cut-off of > 23 spots increment per 300,000 PBMC. Spots increment means GPL-stimulated cultures minus negative controls. Data are presented as median spot increment (or individual value for groups with a single participant) and number (%) of assay-positive and -negative participants. One patient with concomitant MAC and MABC infection is presented separately and was included in the pooled NTM group but not in the individual MAC or MABC subgroup analyses

*MAC Mycobacterium avium* complex; *MABC Mycobacterium abscessus* complex; *NTM* non-tuberculous mycobacteria; *TB* tuberculosis; *CF* cystic fibrosis; *HC* healthy controls

## Discussion

This study demonstrates that glycopeptidolipids (GPLs) derived from *M. avium* elicit significantly stronger IFN-γ responses in patients with pulmonary NTM infections compared to non-infected individuals. These findings support the hypothesis that GPLs are potent drivers of a measurable T-cell-mediated immune response and may represent a promising diagnostic target for NTM infections.

The GPL-IGRA displayed high specificity (98%) but limited and NTM-species-dependent sensitivity. Sensitivity was substantially higher in patients with MAC (64%) than in those with MABC (19%). Consistent with these diagnostic performance characteristics, GPL-induced immune responses were heterogeneous and were significantly higher in MAC than in MABC patients. Thus, using the current cut-off, the assay shows potential to support the diagnosis of an NTM infection, especially for MAC infection. As MAC-derived GPLs were used for stimulation, the better assay performance in MAC patients compared to patients with MABC and other NTM is not surprising. Notably, GPL expression and composition varies considerably among NTM species and even among strains or morphotypes within the same species [4]. In this context, the rather low sensitivity especially for MABC may reflect intrinsic differences in GPL expression or structure between MAC and MABC species, as well as differential host immune recognition. Notably, MABC strains - particularly virulent strains with a rough colony morphotype - often express little to no GPL, which may significantly impair immune recognition and response [5]. In our study, only one participant was identified to have an only rough (GPL-low) colony morphotype MABC isolate, all others with available data had smooth (GPL-rich) or mixed smooth and rough colony morphotypes. Our data therefore do not allow to determine whether the level of GPL expression is associated with the level of in vitro IFN-γ response to GPL stimulation. Importantly, colony morphology is not a universal surrogate for GPL expression across NTM species. Smooth colony morphotypes lacking detectable GPLs have been described and clinically relevant NTM species with these characteristics might escape detection by a GPL-based immune assay [8]. Conversely, the structural characteristics of GPLs may offer opportunities for further assay development. Several GPL-producing mycobacteria share a conserved lipopeptide core, whereas species- and serovar-specific structural diversity results from differences in glycosylation and other modifications [4]. A conserved GPL core structure may therefore represent a potential candidate antigen for a more broadly applicable GPL-based IGRA. Alternatively, species-specific GPL structures could be exploited to develop tailored antigen panels with improved species-level discrimination.

In contrast to established serological tests such as anti-GPL-core IgA detection, which showed moderate sensitivity and specificity for MAC-PD [6], the GPL-IGRA provides a means to directly assess cell-mediated immunity. In our small study we did not find significant differences in immune responses in participants with clinical NTM-PD as compared participants without NTM-PD. Notably, the diagnosis of NTM-PD is difficult and sometimes not definite, especially in CF, where symptoms and radiologic features of CF lung disease significantly overlap with those of NTM-PD. One CF patient without former or current NTM isolation had a positive GPL-IGRA (33 spots increment), possibly indicating a past, spontaneously cleared infection NTM or a failed NTM cultivation. Larger cohorts and longitudinal study designs are needed to address the question of whether there is a role for this assay in distinguishing active disease from colonization, a key clinical challenge in NTM diagnostics [1]. In this context, GPL-IGRAs may also hold potential beyond initial diagnosis. For instance, previous research in paediatric lymphadenitis has shown that GPL-specific responses diminish following successful treatment [7], suggesting a role in monitoring therapeutic response.

In summary, this study confirms the immunogenic properties of mycobacterial GPL and reinforces their diagnostic potential in pulmonary NTM infections, particularly those caused by MAC. Future studies should further exploit both conserved and species-specific features of GPLs by evaluating shared GPL core structures as well as species-specific antigen panels in large cohorts.

## Data Availability

The datasets used and/or analysed during the current study are available from the corresponding author on reasonable request.

## Abbreviations

CF: Cystic fibrosis
GPLs: Glycopeptidolipids
HC: Healthy controls
IGRA: Interferon γ release assays
MABC: Mycobacterium abscessus complex
MAC: Mycobacterium avium complex
NTM: Non-tuberculous mycobacteria
NTM-PD: NTM pulmonary disease
PBMC: Peripheral blood mononuclear cells
TB: Tuberculosis
